# Single-Pixel Imaging in Space and Time with Optically Modulated Free Electrons

**DOI:** 10.1021/acsphotonics.3c00047

**Published:** 2023-04-19

**Authors:** Andrea Konečná, Enzo Rotunno, Vincenzo Grillo, F. Javier García de Abajo, Giovanni Maria Vanacore

**Affiliations:** † ICFO-Institut de Ciencies Fotoniques, The Barcelona Institute of Science and Technology, Castelldefels, Barcelona 08860, Spain; ‡ Central European Institute of Technology, Brno University of Technology, 612 00 Brno, Czech Republic; § Centro S3, Istituto di Nanoscienze-CNR, 41125 Modena, Italy; ∥ ICREA-Institució Catalana de Recerca i Estudis Avançats, Passeig Lluís Companys 23, 08010 Barcelona, Spain; ⊥ Laboratory of Ultrafast Microscopy for Nanoscale Dynamics (LUMiNaD), Department of Materials Science, University of Milano-Bicocca, Via Cozzi 55, 20121 Milano, Italy

**Keywords:** single-pixel imaging, electron microscopy, electron beam shaping, electron−light interaction, ultrafast dynamics

## Abstract

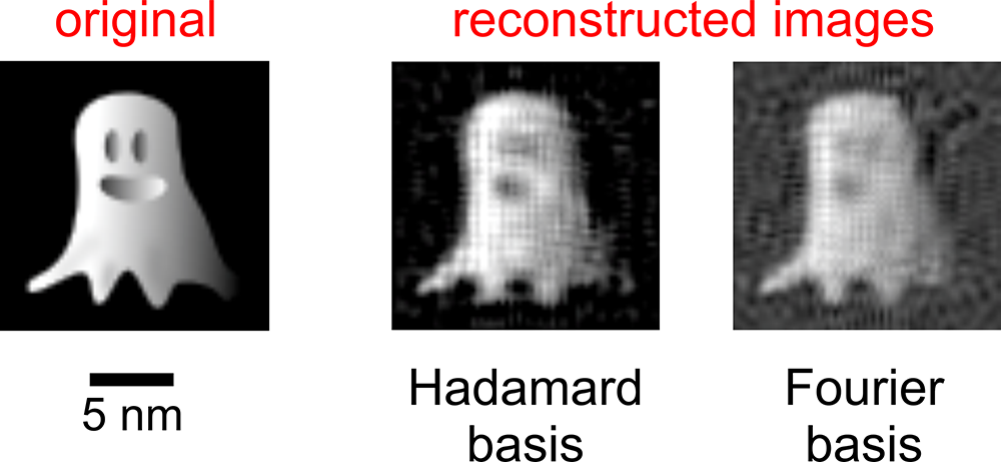

Single-pixel imaging, originally developed in light optics, facilitates fast three-dimensional sample reconstruction as well as probing with light wavelengths undetectable by conventional multi-pixel detectors. However, the spatial resolution of optics-based single-pixel microscopy is limited by diffraction to hundreds of nanometers. Here, we propose an implementation of single-pixel imaging relying on attainable modifications of currently available ultrafast electron microscopes in which optically modulated electrons are used instead of photons to achieve subnanometer spatially and temporally resolved single-pixel imaging. We simulate electron beam profiles generated by interaction with the optical field produced by an externally programmable spatial light modulator and demonstrate the feasibility of the method by showing that the sample image and its temporal evolution can be reconstructed using realistic imperfect illumination patterns. Electron single-pixel imaging holds strong potential for application in low-dose probing of beam-sensitive biological and molecular samples, including rapid screening during in situ experiments.

## Introduction

Single-pixel imaging (SPI) is a key application of structured-wave illumination. This method, which has been recently developed in the context of optical imaging, relies on the interrogation of a certain object using a number of spatially modulated illumination patterns while synchronously measuring the total intensity of the scattered light captured by a single-pixel detector.^1−4^ Key elements in this method are (i) a spatial light modulator (SLM), which provides the spatial encoding of the illumination patterns that is necessary for image reconstruction and (ii) the inherent “sparsity” of typical real-space images such that the bulk of the information is only contained in a limited number of pixels, and consequently, compressed sensing (CS) can be used.^5−8^ CS uses prior knowledge of sparsity in the coefficient domain, making the reconstruction of the image possible by using a smaller number of measurements. Specifically, *O*(*K* log(*N*)) measurements are typically needed if the information is *K*-sparse and has *N* pixels.

The idea behind SPI is to perform a number of sequential measurements with specific illumination patterns expressed on a sufficiently complete basis that can be either incoherent (random patterns) or spatially correlated (such as Hadamard or Fourier bases) with the object to be imaged. The ensemble of *M* measurements, identified by the vector χ, is then correlated to the image *T* (sample transmission function) with a number of pixels *N*_pix_ (in which one usually has *M* ≪ *N*_pix_) through the *M* × *N*_pix_ measurement matrix *H*, which contains the employed SLM patterns, such that χ = *HT*. An image reconstruction algorithm is then used to retrieve a reconstructed image T*.

In optical microscopy, the SPI technique is well-established and its unique measurement scheme has demonstrated far superior performance with respect to conventional imaging. This is because the illumination patterns used for sampling can be custom-tailored to maximize the amount of information acquired during the measurement, whereas in conventional imaging, information gathering is bound to stochastic processes. Different aspects of this idea have been the topic of recent relevant literature in the field of SPI. In particular, several groups have demonstrated that the ordering of Hadamard patterns, for instance, is of primary importance to maximize the effectiveness of CS algorithms. Different orderings based on the significance of the patterns (i.e., different *a priori* knowledge) have been proposed, such as, to mention a few, the “Russian Dolls” ordering,^9^ the “cake cutting” ordering,^10^ the “origami pattern” ordering,^11^ and an ordering based on the total variation of the Hadamard basis.^12^ This concept can be pushed to its ultimate limit when deep learning (DL) is used to gather *a priori* information and identify the best set of illumination patterns.^13^ In this way, it has been demonstrated that, in a limiting scenario in which an object must be identified within a restricted pool of choices, the task can be accomplished without even needing to reconstruct the image,^14^ but just after a single SPI measurement. Incidentally, compressed sensing approaches have recently been used in a transmission electron microscope (TEM) for encoding temporal dynamics in electron imaging with a 10 kHz frame rate (100 μs resolution).^15^

In SPI, the number of illumination patterns required for high-quality imaging increases proportionally with the total number of pixels. However, CS methods and, more recently, DL approaches have been considered to substantially reduce the number of measurements necessary for the reconstruction of an image with respect to the total number of unknown pixels. This is an extremely interesting aspect for electron microscopy since it would entail a lower noise, faster response time, and lower radiation dose with respect to conventional imaging. DL approaches, which have already demonstrated superior performances with respect to CS in terms of speed and sampling ratio, can be organized into three categories: (i) improving the quality of reconstructed images;^16−19^ (ii) identifying the best illumination strategy by exploiting the features learned during training;^13,14^ and (iii) reconstructing the target image directly from the measured signals.^19−23^ Also, a reduction in the sampling rate well below the Nyquist limit (down to 6%) has been demonstrated using DL.

Such advantages would be particularly appealing in the context of electron imaging of nano-objects in their biological and/or chemical natural environment, for which the minimization of the electron dose is critical^24,25^ to avoid sample damage. Initial attempts have been made using MeV electrons with beam profiles controlled by laser image projection on a photocathode.^26^ This method is, however, incompatible with the subnanometer resolution achieved in TEMs through electron collimation stages. Subnanometer resolution for SPI thus requires patterning of high-quality coherent beams. In TEMs, SPI has never been proposed and adopted before, mainly due to the lack of fast, versatile, and reliable electron modulators that would be able to generate the required rapidly changing structured electron patterns.

Here, we propose to implement electron SPI (ESPI) in TEMs by illuminating the specimen using structured electron beams created by a photonic free-electron modulator (here referred as PELM). The PELM is based on properly synthesized localized electromagnetic fields that are able to create an efficient electron modulation for programmable time/energy and space/momentum control of electron beams. Our approach adopts optical field patterns to imprint on the phase and amplitude profile of the electron wave function, an externally controlled well-defined modulation varying both in time and space while the electron pulse crosses the light field. The PELM concept relies on the ability to modulate electrons with optical fields^27−31^ down to attosecond timescales^32−36^ and along its transverse coordinates.^37−40^ In essence, we overcome the problem of designing and fabricating complicated electron optics elements by resorting to shaping light beams, which has been proven a much easier task to perform, while in addition, it enables fast temporal modulation. Indeed, a critical advantage of our approach with respect to existing methods lies on the possibility of achieving an unprecedented ultrafast switching and an extreme flexibility of electron manipulation, which can also open new quantum microscopy applications.^41,42^

A suitable platform for generating the required light field configurations is represented by a light-opaque, yet electron-transparent thin film on which an externally controlled optical pattern is projected from an SLM. The SLM provides an out-of-plane electric field, *E_z_*(*x*,*y*), with a customized transverse configuration that embodies the required laterally changing phase and amplitude profiles. In such a configuration, the spatial pattern imprinted on the incident light field by the SLM is directly transferred onto the transverse profile of the electron wavepacket, as recently shown both theoretically^43,44^ and experimentally.^45,46^ Different portions of the electron wave profile experience a different phase modulation as dictated by the optical pattern. We can thus obtain an externally programmable electron beam with a laterally changing encoded modulation. Moreover, the ability to modulate the electron phase and amplitude has the potential to overcome Poisson noise,^47,48^ which is a key aspect that renders the SPI method not only feasible but also advantageous in terms of low-dose imaging.

A synchronized intensity measurement followed by a CS or DL reconstruction could then be used to retrieve the sample image. Of course, the possibility to use CS or DL algorithms strictly relies on the amount of *a priori* information known about the object under investigation.^7^ This is particularly relevant for ESPI, which can benefit from such *a priori* information, especially in terms of optimal discrimination, more than conventional imaging. In fact, standard TEM imaging is generally object-independent and any *a priori* information is applied only after acquisition to interpret the image, something that can be understood as a denoising procedure. Instead, SPI allows one to optimize the acquisition strategy even before starting the experiment and, thus, holds a direct advantage when using the appropriate pattern basis (see the Supporting Information for a direct example).

**Figure 1:**
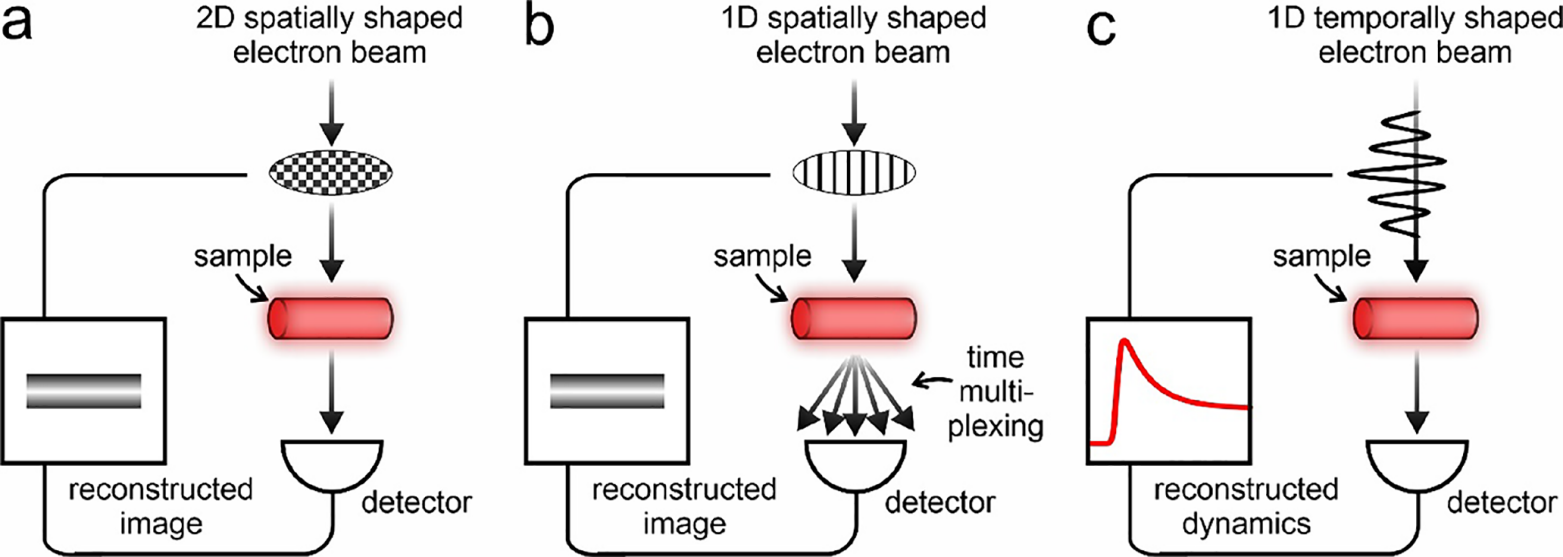
Single-pixel imaging with electrons. Schematic representation of different single-pixel schemes that are amenable to implementation in a transmission electron microscope for 2D spatial imaging (a), 1D spatial imaging (b), and 1D temporal reconstruction (c).

In Figure 1a–c, we present different single-pixel schemes that can be implemented in an electron microscope for 2D spatial imaging (Figure 1a), 1D spatial imaging (Figure 1b), and 1D temporal reconstruction (Figure 1c). Specifically, 2D spatial imaging involves the use of a basis of modulation patterns changing in both transverse directions *x* and *y* (for instance, a Hadamard basis) for full 2D image reconstruction. Instead, 1D spatial imaging involves the use of modulation patterns changing only along one direction (such as a properly chosen Fourier basis) coupled to temporal multiplexing of the electron beam on the detector, which should enable a simpler and faster 1D image reconstruction.

The third scenario of temporal reconstruction is conceptually novel. Importantly, the 1D single-pixel reconstruction algorithm works for any dependent variable of the system phase space. This implies that, by choosing a well-defined basis of temporally changing modulation functions, such as a series of monochromatic periodic harmonics, it would be possible to reconstruct the time dynamics of a sample. The nature of the method would also allow us to reconstruct the dynamical evolution on a temporal scale much smaller than the electron pulse duration because the resolution depends only on the different frequency components of the basis and not on the length of the electron wavepacket. In principle, this approach could even be implemented with a continuous electron beam.

## Results

### Principles of Single-Pixel Imaging

Single-pixel imaging relies on pre-shaped illumination intensity patterns *H^m^*(**R**_S_) that are transmitted through a sampled specimen described by a spatially dependent amplitude transmission function *T*(**R**_S_), defining the sample image, such that the intensity collected at the detector and associated with the *m*^th^ illumination pattern is
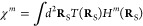
1where we integrate over the sample plane and χ^*m*^ are the elements of the measurement vector. The target is to reconstruct the sample transmission function
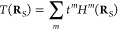
2in terms of coefficients *t^m^*. Now, we assume that the overlap between the illumination patterns is described by
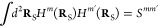
3where *S*^*mm*′^ are real-valued coefficients. Then, by substituting eq 2 in eq 1, we retrieve
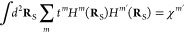
4

We note that if the illumination patterns form an orthonormal basis, then we immediately recover *t^m^* = χ^*m*^ (i.e., the intensities recorded at the detector can directly serve as the expansion coefficients). However, in the general case, where eq 3 produce nonzero nondiagonal elements, the expansion coefficients are
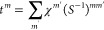
5

By substituting the coefficients back in eq 2, we find the general formula
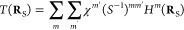
6for the reconstruction of the transmission function of the specimen. It is worth noting that, besides our current choice, many different orthogonalization algorithms have been implemented in the literature (see for instance ref (49)), which can also be used in combination with our ESPI scheme.

### Single-Pixel Imaging in TEM via a Photonic Electron Modulator

**Figure 2:**
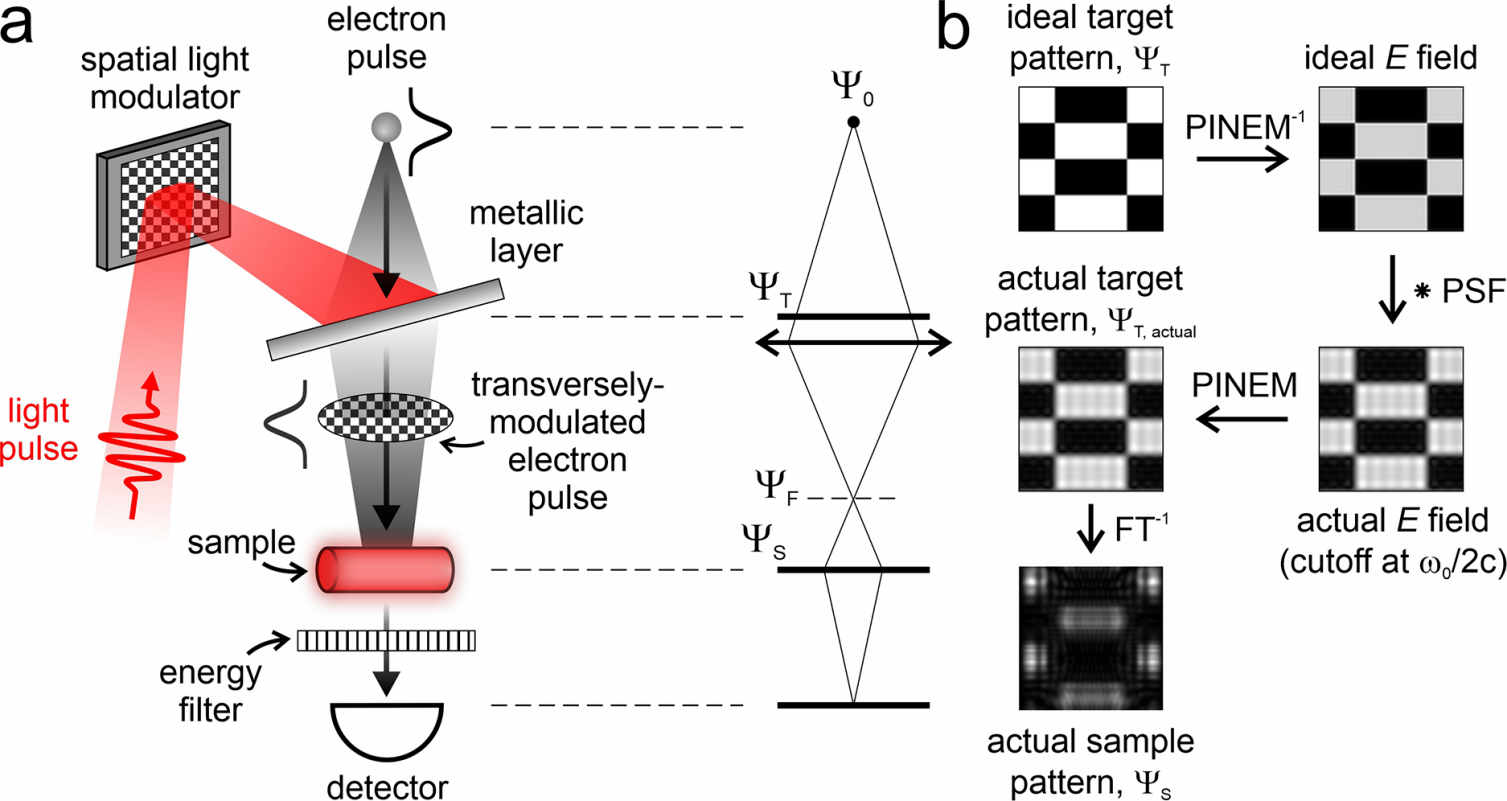
Electron single-pixel imaging (ESPI) via light-mediated electron modulation. (a) Schematic representation of the experimental layout considered for the single-pixel imaging method, implemented by using structured electron beams that are in turn created via light-based manipulation. In our configuration, the spatial pattern imprinted on the incident light field by a programmable spatial light modulator is transferred on the transverse profile of the electron wavepacket by electron–light interaction. (b) Sequence of operations used to calculate the transverse distribution of the electron beam arriving on the sample either when starting from an ideal target pattern or when considering realistic non-ideal conditions. We take a pattern from a Hadamard basis for this example.

We now proceed to analytically describe the scheme utilized to implement the SPI method in an electron microscope. This is shown in Figure 2, where the sample illumination is performed using structured electron beams created via light-induced manipulation. Efficient and versatile phase and intensity modulation of a free electron can be achieved using a PELM device. In our configuration, the spatial pattern imprinted on the incident light field by a programmable SLM is transferred on the transverse profile of the electron wavepacket by electron–light interaction.^45^ This is generally dubbed as the photon-induced near-field electron microscopy (PINEM) effect,^27,28,50^ although in our configuration, we actually exploit the breaking of translational symmetry induced by a thin film (inverse transition radiation), as described in detail in refs (35)(51), and (52), rather than a confined near field induced by a nanoscale structure. The shaped electron wavepacket is then propagated through the TEM column toward the sample.

The electron–light interaction under consideration admits a simple theoretical description:^35,43^ starting with an electron wave function ψ_0_ incident on the PELM, after the interaction with the light field has taken place, the electron wave function is inelastically scattered into mutually coherent quantized components of amplitude
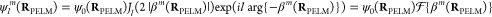
7corresponding to electrons that have gained () or lost ()  quanta of photon energy ℏω. Here,  represents the PINEM operator, which depends on the imprinted variation of the transverse profile, governed by the coupling coefficient
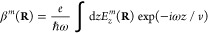
8where *ℏ* is the reduced Planck constant, *e* is the elementary charge, and the light illumination is further characterized by the electric field **E***^m^* (see the Supporting Information for a detailed calculation of β^*m*^ in a metallic thin film). We assume that beam electrons have velocity **v** ∥ *ẑ*. Due to the inelastic nature of the PINEM interaction, post-interaction electrons gain or lose different numbers of quanta, associated with kinetic energy changes . In addition, the corresponding contributions to the wave function in eq 7 have different spatial distributions of amplitude and phase. For our purpose, it would be beneficial to place a simple energy filter after the PELM, selecting, for example, the  component only (i.e., electrons gaining one photon energy quantum).

The energy filter needs to efficiently separate a given sideband of the electron energy distribution from the rest of the spectrum. The higher the filter efficiency, the larger the contrast in the modulation pattern, also resulting in a more reduced noise in the final image. However, a relatively modest reduction should be sufficient as we estimate that ∼34% of the electron signal can be placed in the first (gain or loss) sideband. In addition, as we are interested in intensity patterns, the first gain or loss sidebands both deliver the same pattern, and thus, 68% of the electrons are contributing by simultaneously filtering both bands. As a possible improvement, light patterns could be also engineered to eventually remove the need for energy filtering. These possibilities are in fact enabled by properly tuning the light field intensity and, thus, the resulting modulation of the electron beam and its energy distribution.^35^

A practical approach toward the design of the structured beam sample illumination is to define a suitable  and thus also β^*m*^, study the propagation of the wave function to the plane of the specimen, and then find optimal settings for the aperture size, beam energy, and focal distance in such a way that *H^m^*(**R**_S_) mimics the optical illumination pattern. For ESPI, we thus impose the inelastically scattered electron wave function, , to be equal to the target pattern, ψ_*T*_, defined within the chosen basis ( = ψ_*T*_). Once this is defined, we can retrieve the coupling coefficient, β^*m*^, and, therefore, the light field, *E*_*z*_^*m*^, to be implemented on the SLM by applying an inverse PINEM transformation, , to the target pattern ψ_*T*_ (see Figure 2 and also Figure S1 for a Hadamard basis and Figure S2 for a Fourier basis).

Particularly important is to demonstrate the feasibility of the method also under realistic, non-ideal conditions. We do this by applying a momentum cutoff (ω_0_/*nc*, where *n* = 1,2, and 3) on the retrieved light field defined by a momentum-dependent point spread function (PSF) to take into account the finite illumination wavelength and limited numerical aperture. This produces the actual light field, *E*_*z*_^*m*^|_actual_, from which we can calculate the actual coupling coefficient, β^*m*^|_actual_. By applying the PINEM transformation, , we can in turn find the actual target pattern, ψ_*T*_|_actual_.

The sequence of operations is defined in eq 9 below and visually shown in Figure 2 and Figures S1 and S2:
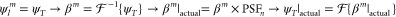
9

To maximize the efficiency of the electron amplitude and phase modulation, it is beneficial to place the PELM onto a plane along the microscope column where the beam is extended to diameters much larger than the wavelength of the optical illumination. In such a scenario, we can achieve the desired detail in the variation of the transverse wave function profile. However, we then have to rely on electron lenses to focus the beam on the sample.

The focusing action together with the free propagation of the electron wave function between the PELM and the sample planes is described, within the paraxial approximation, as^43^
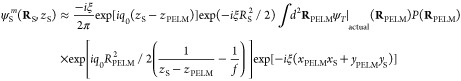
10where we have defined ξ = *q*_0_/(*z*_S_ – *z*_PELM_) with *q*_0_ as the electron wave vector that varies with acceleration voltage and the coordinates **R**_S_ = (*x*_S_, *y*_S_) evolving in the sample *z* = *z*_S_ plane. In addition, *P*(**R**_PELM_) is a transmission (pupil) function, which becomes 1 if the electron beam passes through an effective aperture placed in the PELM plane and 0 otherwise. We have also replaced the focusing action of all subsequent lenses by a single aberration-free thin lens with a focal distance *f* placed virtually just after the PELM. The illumination intensity at the sample resulting from eq 10 is
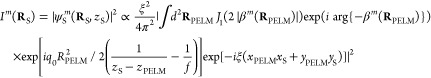
11

In Figure 2b and Figures S1 and S2, we show the realistic sample patterns obtained for a Hadamard pattern and a Fourier pattern, chosen as examples when using the following parameters: 200 keV electrons, lens focal distance *f* = 1 mm, *z*_PELM_ = 0, *z*_S_ = 1.0008 mm (defocus of 0.8 μm), and PELM area of 10 × 10 μm^2^.

It is important to mention that the ESPI method here proposed is based on electron intensity modulation, rather than phase modulation. Therefore, there are no stringent constraints or requirements on the transverse coherence of the electron beam for the method to work properly. This is what makes this technique readily available in many different experimental configurations where, for instance, one would favor electron current density over coherence to increase the signal-to-noise ratio of the measurements. Of course, if the transverse coherence of the electron beam is commensurate with the spatial scale at the PELM plane in which a significant phase change of the interaction strength β^*m*^ takes place, then phase modulation effects could be visible. Under such conditions, the method could take advantage of the possibility to imprint also a phase modulation—besides an amplitude modulation—on the electron transverse profile. This aspect would not only largely increase the number of patterns forming the basis used for the reconstruction, but it could also potentially allow us to image phase objects via the ESPI method in analogy to optical SPI.^53^

An efficient reconstruction can be achieved with a binary illumination using the Hadamard basis, where *N* sample pixels (e.g., a set of discrete **R**_S_ points) can be reconstructed with *N* patterns.^54^ However, because the Hadamard basis adopts +1 and −1 values to ensure orthogonality, in our case, non-orthonormality issues might arise from the fact that we are working with intensity patterns that are never negative. This aspect, together with the imperfect illumination under realistic, non-ideal conditions (see Figure 2), implies that the actual sample patterns no longer represent an orthonormal basis, and therefore, the reconstructed sample transmission function, *T*(**R**_S_), has to the corrected as described in eqs 5 and 6 via the overlap matrix *S*^*mm*′^. The latter and its inverse are shown in Figure S3 for the Hadamard basis. Another option for a basis is to use Fourier-like intensity patterns (Fourier basis), which are defined as
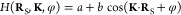
12where **K** are spatial frequencies, *a* and *b* are constants, and φ is a phase.

## Discussion

### Image Reconstruction Using Hadamard and Fourier Bases

We show next several examples of image reconstruction using different bases. For illustration, we consider a Siemens star and a ghost image. The former is a binary {0,1} image with sharp transitions, whereas the latter presents small features, is asymmetric, and shows a gradual intensity variation from 0 to 1. This allows us to test in full the capabilities of the method.

**Figure 3:**
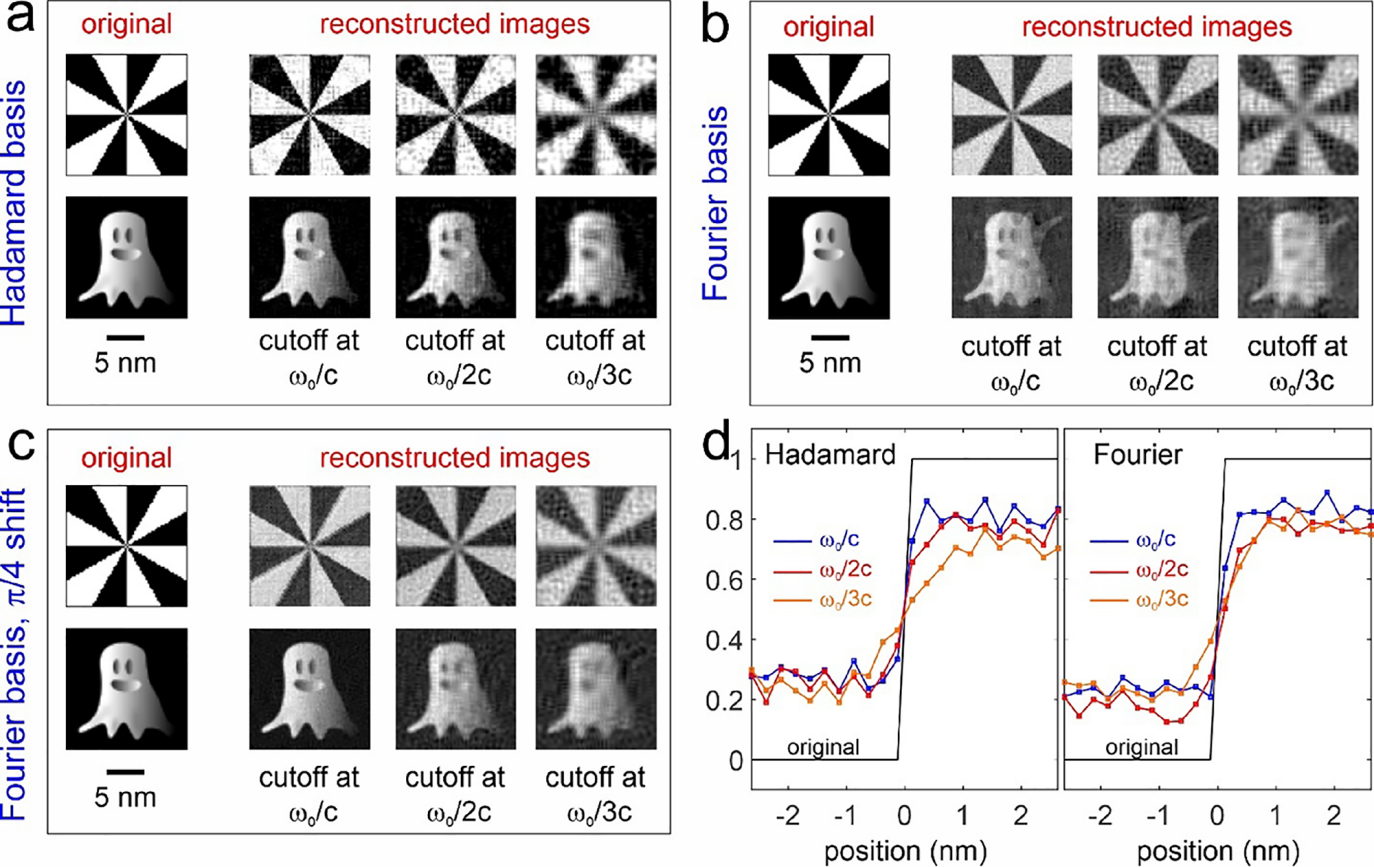
ESPI imaging using Hadamard and Fourier bases. We discuss image reconstruction of a Siemens star and a ghost image performed using a Hadamard basis (a), Fourier basis (b), and Fourier basis with a π/4 phase shift (c). Reconstructed images are shown for different momentum cutoffs (ω_0_/*nc*, where *n* = 1,2, and 3) on the retrieved light field. The field of view of all images if 16 × 16 nm^2^. (d) Spatial profiles obtained at the sharp edge of the Siemens star when using a Hadamard basis (left) and a Fourier basis (right). The black curve represents the original image, while the blue, red, and orange curves are associated with frequency cutoffs of ω_0_/*c*, ω_0_/2*c*, and ω_0_/3*c*, respectively. The spatial resolution is estimated by taking the 10:90 value of the error function fit for each curve. We obtain the following resolutions: 0.29 nm at a cutoff of ω_0_/*c*, 0.49 nm at a cutoff of ω_0_/2*c*, and 1.43 nm at a cutoff of ω_0_/3*c* for the Hadamard-reconstructed images; 0.25 nm at a cutoff of ω_0_/*c*, 0.63 nm at a cutoff of ω_0_/2*c*, and 1.01 nm at a cutoff of ω_0_/3*c* for the Fourier-reconstructed images.

In Figure 3a, we plot the ideal and reconstructed Siemens star and ghost image considering different cutoffs for a Hadamard basis. Clearly, the reconstructions reproduce all the main features of the original images, although we also encounter some noise and even a few negative values, which should not appear. The latter are due to ill-conditioned matrix inversion that we need to use for the reconstruction to compensate for the non-orthonormality of the involved patterns. In Figure 3b, we plot the results of image reconstruction using a Fourier basis. Although for the Siemens star reconstruction, the Fourier basis is performing similarly as the Hadamard basis, for the ghost image, it is clear that the Fourier basis with the same number of patterns (64 × 64) yields artifacts: a faint mirror-reflected ghost is superimposing on the actual one. The reconstruction with the Fourier basis becomes considerably better when taking into account a phase offset so that the Fourier pattern would no longer be symmetric with respect to the origin. In Figure 3c, we consider an offset of φ = π/4 for the corresponding reconstructed sample images. As a result, the reconstructed ghost image no longer exhibits the faint mirror-reflected artifact that was visible in Figure 3b.

Based on the results of Figure 3, we perform additional quantitative analysis on the images to compare the different reconstruction algorithms and bases. We extract the peak signal-to-noise ratio (PSNR) for both the Siemens star and ghost images for the two bases and three different cutoff frequencies used. From these calculations, we conclude that the reconstruction with a Fourier basis provides values of the PSNR about 10% better than the Hadamard basis for all cutoffs. This is probably due to the fact the Hadamard basis is composed of binary patterns, which are extremely sensitive to distortions caused by diffractive effects during electron propagation, whereas such effects are mitigated for Fourier patterns, which are characterized by gradual, smooth variations. The better quality of the images reconstructed via Fourier patterns directly implies a better image resolution. This is visible in Figure 3d, where we show the effect of the reconstruction on the spatial shape of a particularly sharp feature of the Siemens star. As expected, we observe an increasing broadening when smaller cutoff frequencies are considered. The estimated spatial resolution (for a 10:90 fit of the error function) varies from 0.29 nm at a cutoff of ω_0_/*c* to 1.43 nm at a cutoff of ω_0_/3*c* for the Hadamard-reconstructed images, whereas the Fourier basis provides slightly better values ranging from 0.25 nm at a cutoff of ω_0_/*c* to 1.01 nm at a cutoff of ω_0_/3*c*.

It is important to mention that the ESPI method that we propose here is intended to be applied to imaging amplitude objects. In fact, in TEMs, a huge amount of information is contained in amplitude-contrast mechanisms, such as mass-thickness contrast, Z-contrast, and bright-field and dark-field imaging as well as electron energy-loss spectroscopy (EELS) and energy-dispersive X-ray spectroscopy (EDX). In a standard TEM, single-pixel detectors are in fact already present. This is for instance the case of the high-angle annular dark-field (HAADF) detector used for performing Z-contrast imaging in STEM mode, which can also provide an experimental verification of the proposed configurations. Besides their use as single-pixel detectors, STEM detectors are also able to gather signals in different angular regimes. Such capability is generally used to access simultaneously more information about the sample (typically chemical information). In the SPI context, we can anticipate a more complex partition of the detector—exploiting its angular detection capability—bridging the gap with other techniques such as integrated differential phase contrast (iDPC) or ptychography.

### Temporal Electron Single-Pixel Imaging

**Figure 4:**
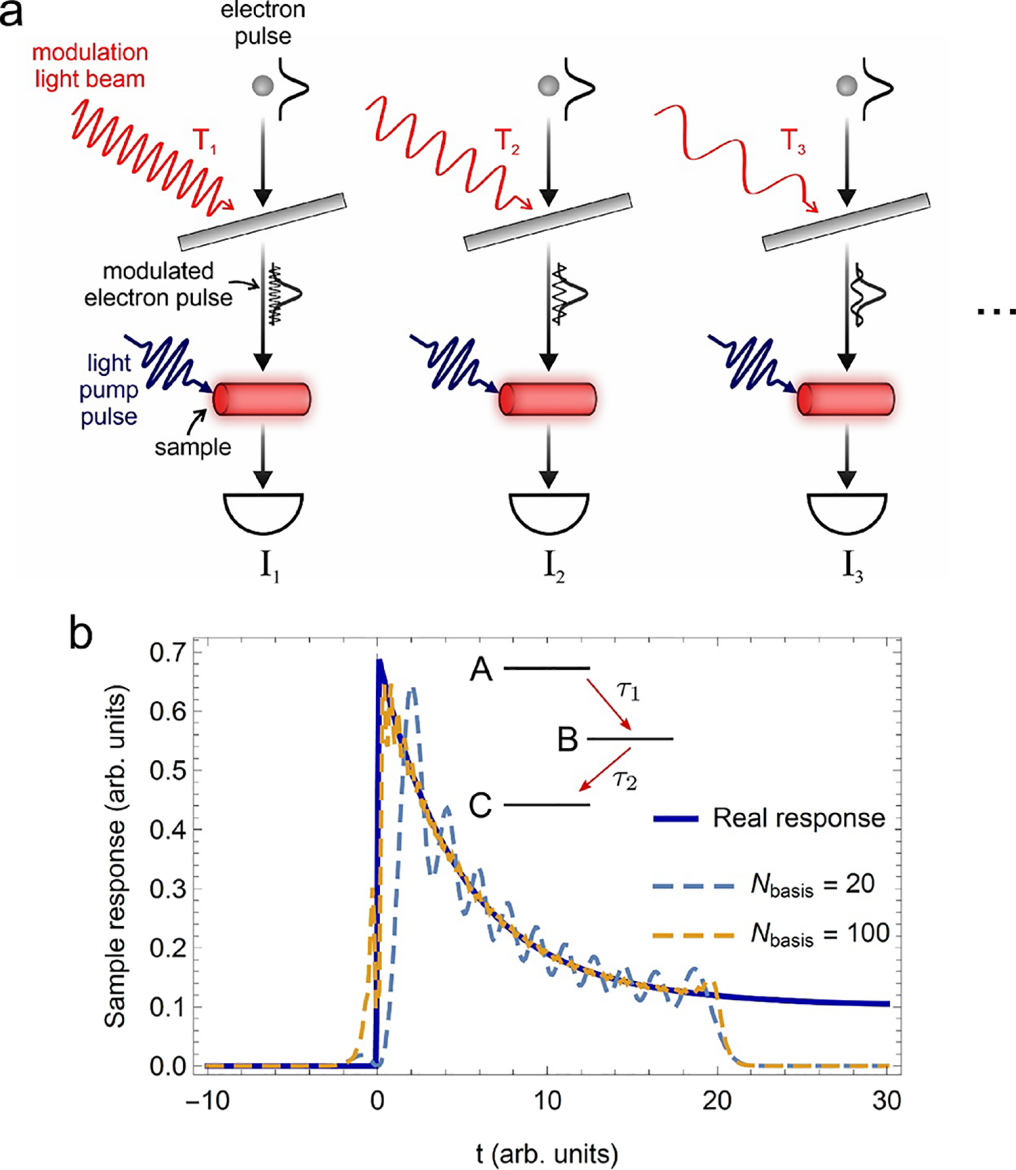
Temporal electron single-pixel imaging. 1D temporal single-pixel reconstruction of a material dynamics. (a) A sequence of long light pulses with varying periods *T_j_* couple to the electron pulse via inverse transition radiation mediated by a metallic plate. We show three different periods: *T*_1_ < *T*_2_ < *T*_3_. The longitudinally modulated electron pulse then interacts with the sample in its excited state, and for each period *T_j_*, a scattered intensity *I_j_* is measured. The full temporal evolution of the sample is finally reconstructed from a Fourier-like transformation of the measured signals (see main text for details). (b) Simulated temporal dynamics of a system comprising three states (A, B, and C) according to the diagram in the inset. In the plot, the real response of the system (blue curve), obtained from a rate equation model, is compared with the results of temporal Fourier reconstructions using either 20 basis functions (dashed blue curve) or 100 basis functions (dashed orange curve), as defined in eq S16.

As a final aspect, we present a possible implementation of the 1D temporal ESPI reconstruction scheme. The basic idea is to be able to reconstruct the dynamic behavior of a specimen—for instance, its dielectric response to an optically induced electronic excitation—using a sequence of temporally modulated electron pulses with varying periodicity. In Figure 4a, we show the schematics of the experiment, where a sequence of long light pulses with varying periods *T_j_* couple to the electron pulse via inverse transition radiation as mediated by the aforementioned metallic plate. The longitudinally modulated electron pulse then interacts with the sample in its excited state, and for each period *T_j_*, a signal *I_j_* is measured. In terms of the single-pixel formalism, this means that we are choosing a one-dimensional Fourier-like basis for the evolution of the incident electron current as a function of delay time with respect to the pumping time:
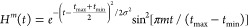
13where *t*_min_ and *t*_max_ determine the boundaries of the sampling time interval and σ^2^ is the variance of the envelope of the probing electron wave function.

As discussed in detail in the Supporting Information, we have simulated the dynamics of a system comprising three states (A, B, and C) according to the diagram in Figure 4b. At time zero, the system is taken to be pumped to an excited state A, from which it decays in a cascade fashion to B and then to C. The time evolution of the populations of the three states within our model system is governed by three rate equations. In Figure 4b, we show the results of a temporal Fourier reconstruction using the basis functions defined in eq 13 in an analogous way to the spatial domain and, again, taking into account the non-orthogonality of the illumination basis. We demonstrate that, already with 20 basis functions, the gross features of the temporal response of the system are retrieved.

It is important to note that the temporal resolution of the measurement no longer depends on the duration of the electron and light pulses but only on the frequency bandwidth of the light field used for electron modulation. This aspect is extremely interesting because it opens the possibility of using continuous electron and light beams, provided that an efficient electron–light coupling is achieved.^55−59^ A possible technological implementation of such a scheme can be realized by using an optical parametric amplifier (OPA) coupled to a difference frequency generator (DFG). This type of configuration would provide light fields with periods in the 0.8–50 fs range, making our approach invaluable to investigate sample dynamics with a temporal resolution that is far below that of state-of-the-art ultrafast electron microscopy, and equally combined with the atomic spatial resolution provided by electron beams.

## Conclusions

In this work, we have proposed the implementation of single-pixel imaging in electron microscopy and predicted that such a method can provide image reconstruction with subnanometer resolution as well as temporal dynamics reconstruction with a precision of a few femtoseconds while benefiting from *a priori* information, especially in terms of optimal discrimination. This potential is examined here when using fast and versatile optically induced electron beam modulation, although it can also be applied to other schemes of electron beam shaping using, for example, electrostatic and magnetostatic devices.^60−64^ Finally, the possibility of using deep learning approaches in the reconstruction algorithm can substantially reduce, by more than one order of magnitude,^18^ the number of measurements necessary to form an image, thus making such a method suitable for high-spatiotemporal-resolution, low-dose probing of beam-sensitive biological and molecular samples.

## Supporting Information Available

Section S1: Determination of the coupling coefficient β for a homogeneous thin film; Section S2: detailed description of the analytical calculations behind the temporal electron single-pixel imaging approach; Section S3: additional notes on optimal discrimination and use of *a priori* information in single-pixel imaging and conventional imaging (PDF)
