# Supplementary material for: Single-Pixel Imaging in Space and Time with Optically Modulated Free Electrons
Source: ACS Photonics. 2023 Apr 19;10(5):1463–72. doi: 10.1021/acsphotonics.3c00047 (PMC10197172; doi:10.1021/acsphotonics.3c00047)
Supplement: Supplementary file 1 — ph3c00047_si_001.pdf [file ph3c00047_si_001.pdf]

SUPPORTING INFORMATION

Single-Pixel Imaging in Space and Time with Optically-Modulated Free Electrons

Andrea Konečná[1,2], Enzo Rotunno[3], Vincenzo Grillo[3], F. Javier García de Abajo[1,4,*], and Giovanni Maria Vanacore[5,*]

1. ICFO-Institut de Ciencies Fotoniques, The Barcelona Institute of Science and Technology 08860 Castelldefels (Barcelona), Spain
2. Central European Institute of Technology, Brno University of Technology, 612 00 Brno, Czech Republic
3. Centro S3, Istituto di Nanoscienze-CNR, 41125 Modena, Italy
4. ICREA-Institució Catalana de Recerca i Estudis Avançats, Passeig Lluís Companys 23, 08010 Barcelona, Spain
5. Laboratory of Ultrafast Microscopy for Nanoscale Dynamics (LUMiNaD), Department of Materials Science, University of Milano-Bicocca, Via Cozzi 55, 20121 Milano (Italy).

* To whom correspondence should be addressed:
javier.garciadeabajo@nanophotonics.es, giovanni.vanacore@unimib.it

S1. Coupling Coefficient Beta for a Homogeneous Thin Film

The coupling coefficient β [see Eq. (8) in the main text] for a superposition of *p*-polarized light waves impinging from the $z<0$ region with angle θ relative to the normal *z* direction on a self-standing homogeneous thin film of thickness d can be written

$$\beta^m(\mathbf{R}) = \int_{-k_0}^{k_0} dk_x \int_{-k_0}^{k_0} dk_y\, exp(i\mathbf{K}\cdot\mathbf{R})\beta^m_{\mathbf{K}}, \tag{S1}$$

which incorporates the fact that, due to the finite wavelength of light $\lambda = 2\pi/k_0 = 2\pi c/\omega$, we can imprint patterns with a precision limited by diffraction (i.e., the integral over transverse light wave vectors $\mathbf{K} = (k_x, k_y)$ is limited to the range $K < k_0$). The contributions of the different transverse wave vector components are

$$\beta^m_{\mathbf{K}} = \frac{ieK}{\hbar\omega k_0}\alpha_{\mathbf{K}}\left[\frac{1}{\omega/v - k_z} + \frac{r_p}{\omega/v + k_z} - \frac{t_p \exp(-i\omega d/v)}{\omega/v - k_z} + A\frac{\exp[i(-\omega/v + k_z')d] - 1}{\omega/v - k_z'} + B\frac{\exp[-i(\omega/v + k_z')d] - 1}{\omega/v + k_z'}\right]. \tag{S2}$$

Here d is the film thickness, while the coefficients $\alpha_{\mathbf{K}}$ are controlled through the proper settings of the spatial light modulator, and r_p, t_p, A, and B are given by

$$r_p = r_p^0 \left[1 - \frac{\left(t_p^0\right)^2 (k_z'/k_z) \exp(2ik_z'd)}{1 - \left(r_p^0\right)^2 \exp(2ik_z'd)}\right], \tag{S3}$$

$$t_p = \frac{\left(t_p^0\right)^2 (k_z'/k_z) \exp(ik_z'd)}{1 - \left(r_p^0\right)^2 \exp(2ik_z'd)}, \tag{S4}$$

$$A = \frac{1}{\sqrt{\epsilon}} \frac{t_p^0}{1 - \left(r_p^0\right)^2 \exp(2ik_z'd)}, \tag{S5}$$

$$B = \frac{1}{\sqrt{\epsilon}} \frac{-t_p^0 r_p^0 \exp(2ik_z'd)}{1 - \left(r_p^0\right)^2 \exp(2ik_z'd)}, \tag{S6}$$

where r_p^0 and t_p^0 are the Fresnel reflection coefficients expressed in terms of the permittivity of the film material ϵ as

$$r_p^0 = \frac{\epsilon k_z - k_z'}{\epsilon k_z + k_z'}, \tag{S7}$$

$$t_p^0 = \frac{2\sqrt{\epsilon} k_z}{\epsilon k_z + k_z'}, \tag{S8}$$

with out-of-plane light wave vector components k_z and k_z' given by

$$k_z = \sqrt{k_0^2 - K^2}, \tag{S9}$$

$$k_z' = k_0 \sqrt{\epsilon - K^2/k_0^2}. \tag{S10}$$

In the perfect-electric-conductor (PEC) limit for the film material, we have $r_p = r_p^0 = 1$ and $t_p = 0$, so the **K**-dependent amplitudes of the coupling coefficient reduce to

$$\beta_{\mathbf{K},\mathrm{PEC}}^m = \frac{2ieK}{\hbar k_0 v} \frac{\alpha_{\mathbf{K}}}{(\omega/v)^2 - (k_z)^2}. \tag{S11}$$

S2. Temporal Electron Single-Pixel Imaging

We consider a system comprising three states (*j* = A, B, and C) according to the diagram in Fig. 4b of the main text. At time zero, the system is taken to be pumped to an excited state A, from

which it decays in a cascade fashion to B and then to C. The time evolution of the populations of the three states within our model system, $p_{\mathrm{A}}, p_{\mathrm{B}}$ and p_{C} (shown in the inset of Fig. 4d), is governed by the rate equations

$$\dot{p}_{\mathrm{A}} = -\frac{p_{\mathrm{A}}}{\tau_1}, \qquad \dot{p}_{\mathrm{B}} = \frac{p_{\mathrm{A}}}{\tau_1} - \frac{p_{\mathrm{B}}}{\tau_2}, \qquad \dot{p}_{\mathrm{C}} = \frac{p_{\mathrm{B}}}{\tau_2}, \tag{S12}$$

which are supplemented by the initial conditions $p_{\mathrm{A}}(0) = 1$, $p_{\mathrm{B}}(0) = p_{\mathrm{C}}(0) = 0$. Here, τ_1 and τ_2 are the lifetimes associated with the decay from A to B and from B to C, respectively. The solution to this set of equations is

$$\begin{aligned} p_{\mathrm{A}} &= e^{-\frac{t}{\tau_1}}, \\ p_{\mathrm{B}} &= \frac{1}{\frac{\tau_1}{\tau_2} - 1}\left(e^{-\frac{t}{\tau_1}} - e^{-\frac{t}{\tau_2}}\right), \\ p_{\mathrm{C}} &= \frac{1}{\frac{\tau_1}{\tau_2} - 1}\left(-\frac{\tau_1}{\tau_2} e^{-\frac{t}{\tau_1}} + e^{-\frac{t}{\tau_2}}\right) + 1. \end{aligned} \tag{S12}$$

We then consider the time evolution of the measured scattering intensity I for electrons interacting with the system at time *t*:

$$\begin{aligned} I(t) &= \Theta(t) \sum_{j \in \{\mathrm{A,B,C}\}} p_j(t) a_j = \\ &= \Theta(t) \left\{ a_{\mathrm{C}} + e^{-\frac{t}{\tau_1}} \left[a_{\mathrm{A}} + \frac{1}{\frac{\tau_1}{\tau_2} - 1} \left(a_{\mathrm{B}} - \frac{\tau_1}{\tau_2} a_{\mathrm{C}} \right) \right] + e^{-\frac{t}{\tau_2}} \frac{a_{\mathrm{C}} - a_{\mathrm{B}}}{\frac{\tau_1}{\tau_2} - 1} \right\}, \end{aligned} \tag{S14}$$

where $\Theta(t)$ is the Heaviside function and the constants a_j are the intensities observed when the system is in state *j*. This expression implicitly assumes that the interaction time per electron is short compared with the decay times.

We use Fourier-like basis functions for the evolution of the incident electron current as a function of time with respect to the pumping time:

$$H^m(t) = e^{-\frac{\left(t - \frac{t_{\max} + t_{\min}}{2}\right)^2}{2\sigma^2}} \sin^2[\pi m\, t/(t_{\max} - t_{\min})], \tag{S15}$$

where $t_{\min}$ and $t_{\max}$ determine the boundaries of the sampling time interval and σ^2 is the variance of the envelope of the probing electron wave function. In our example, we set $\sigma = 0.1(t_{\max} - t_{\min})$, $a_{\mathrm{A}} = 0.7, a_{\mathrm{B}} = 0.2$, $a_{\mathrm{C}} = 0.1$, $\tau_1 = 4$, and $\tau_2 = 8$, all in arbitrary units. The reconstruction is then performed in an analogous way to the spatial domain. We again consider the non-orthogonality of the illumination basis.

In Fig. 4b, we show the reconstructed time profile in the defined interval using either 20 or 100 basis functions, which are able to correctly retrieve the real response of the system. From an

experimental viewpoint, this approach can be used to retrieve the transient dynamics of a material in the few-femtosecond range even when using very long electron pulses (picosecond or longer).

S3. Notes on Optimal Discrimination and Use of *a Priori* Information in SPI and Conventional Imaging

We provide here a more specific example of how *a priori* information can benefit ESPI, especially in terms of optimal discrimination, but less so for post-processing in conventional raster-scanning imaging. For instance, the knowledge that a given sample is sparse in real space (e.g., a few particles dispersed in a large area) is already used in ESPI, in which each measurement of a projection on a given base function gives more information than in conventional imaging, where many measurements of empty pixels are needed before some nonzero signal is detected.

In general, it is non-trivial to make a direct comparison between the two techniques. Conventional raster scanning has the advantage of its simplicity and direct interpretation. However, we identify here a specific example that illustrates some conditions under which ESPI holds a direct advantage.

The key is the use *a priori* information. Raster scanning is assumed to be done with a strategy that is object-independent, while SPI allows one to optimize the acquisition strategy even before starting the experiment. In raster scanning any *a priori* information is applied only after acquisition to interpret the image, something that for conventional imaging can be regarding as de-noising.

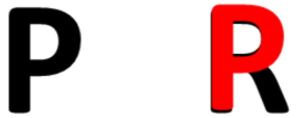

In this comparison we push the condition to the extreme by considering a very strong *a priori* information, and thus, we are left with only two possibilities. Let's consider that our amplitude sample looks like a letter 'P' or a letter 'R', as in the figure above. The difference between the two is in the right-hand leg of the 'R', while the rest of the structure is identical. Standard raster-scanning must necessarily cover also the 'P', as it does not use, by definition, any *a priori* information before performing the experiment. Raster scanning is therefore intrinsically less efficient, and no post-acquisition strategy can remedy the loss of counts in the 'P' body (i.e., a region that does not add anything to the known information). While ESPI could solve the ambiguity with a single electron, raster-scanning could easily be non-conclusive even when using a few of them.

In a recent paper by Troiani *et al*. [Phys. Rev. A **102**, 043510 (2020)], the authors have highlighted that, in order to optimize an electron measurement, the detection must concentrate on the projection on the differences between the two states. In this simplified case, the optimization would thus just concentrate on the shape difference because intensity levels are binary. In the P-R discrimination, the difference is just the right-hand "leg" of the R and it is a 0 or 1 discrimination.

SPI would thus use this *a priori* information to reduce the amount of patterns and electrons used in the reconstruction, whereas conventional imaging would still scan over the entire image and use such *a priori* information only for a post-acquisition de-noising.

To convey a more general argument, we can also consider objects with different levels of transparency (and not only 0 and 1 as in the previous example), where an optimization strategy would be more complex. Let's assume, for instance, that the sample is composed of two parts and, as before, our *a priori* information is very strong, so we are left with only two possible samples to discriminate. For the sample S1 (see figure below), the transmittance is 0.75 in the left part and 0.25 in the right part. The second sample S2 is instead the opposite: 0.25 transmittance on the left and 0.75 transmittance on the right. Once again both samples are only amplitude objects.

Sample S1 Sample S2

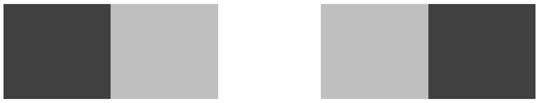

The optimal measurement theory (see, for instance, Troiani et al., Phys. Rev. A **102**, 043510 (2020)) indicates that the ideal measurement relies on the projection (scalar product) on a vector that would produce a difference between the two objects (i.e., the vector **v** = (1,-1) in this case). We assume here that the result of two measurements in the two parts can be summed together (and that the phase of the wave addressing the two parts is controllable). This is experimentally possible only for a SPI scheme in which the detector is in a diffraction plane.

For a experiment, we assume that we can only measure the intensity or amplitude of such projection. Then, the absolute value of the projection of the sample S1 on the vector **v** would give |0.75-0.25| = 0.5, and for the sample S2 this projection would also be |0.25-0.75| = 0.5. This is the same value in the two cases. The optimal discrimination theory thus tells us that the best projector vector can be constructed as **v'** = **v** + (0.5,0.5), and therefore, the two projections for the samples S1 and S2 on the vector **v'** will now be 1 and 0, respectively. Namely, using this projector we would have a “1 or 0” type of experiment even if the transmissivity is not binary in real space. A fundamental reason for this is that SPI is compatible with an optimization in a multiplicity of bases, while raster scanning can only be used to optimize in real space.

THREE-STEP ALGORITHM WITH FOURIER BASIS

When using a Fourier basis, the reconstruction can be alternatively performed with a three-step (or alternatively four-step) algorithm (see Ref. 33 in the main text), where we define

$$\Delta_{\varphi}(\mathbf{K}) = \int \mathrm{d}^2\mathbf{R_S} T(\mathbf{R_S}) H(\mathbf{R_S}, \mathbf{K}, \varphi). \tag{S16}$$

Then, we calculate

$$\Delta_{\mathrm{Tot}} = \left(2\Delta_0 - \Delta_{2\pi/3} - \Delta_{4\pi/3}\right) + i\sqrt{3}\left(\Delta_{2\pi/3} - \Delta_{4\pi/3}\right) \tag{S17}$$

and finally perform the inverse Fourier transform to reconstruct the sample transmission function

$$T(\mathbf{R_S}) = \mathrm{FT}^{-1}\{\Delta_{\mathrm{Tot}}(\mathbf{K})\}, \tag{S18}$$

which can be done after collecting the intensities at the detector, obtained with varying spatial frequencies.

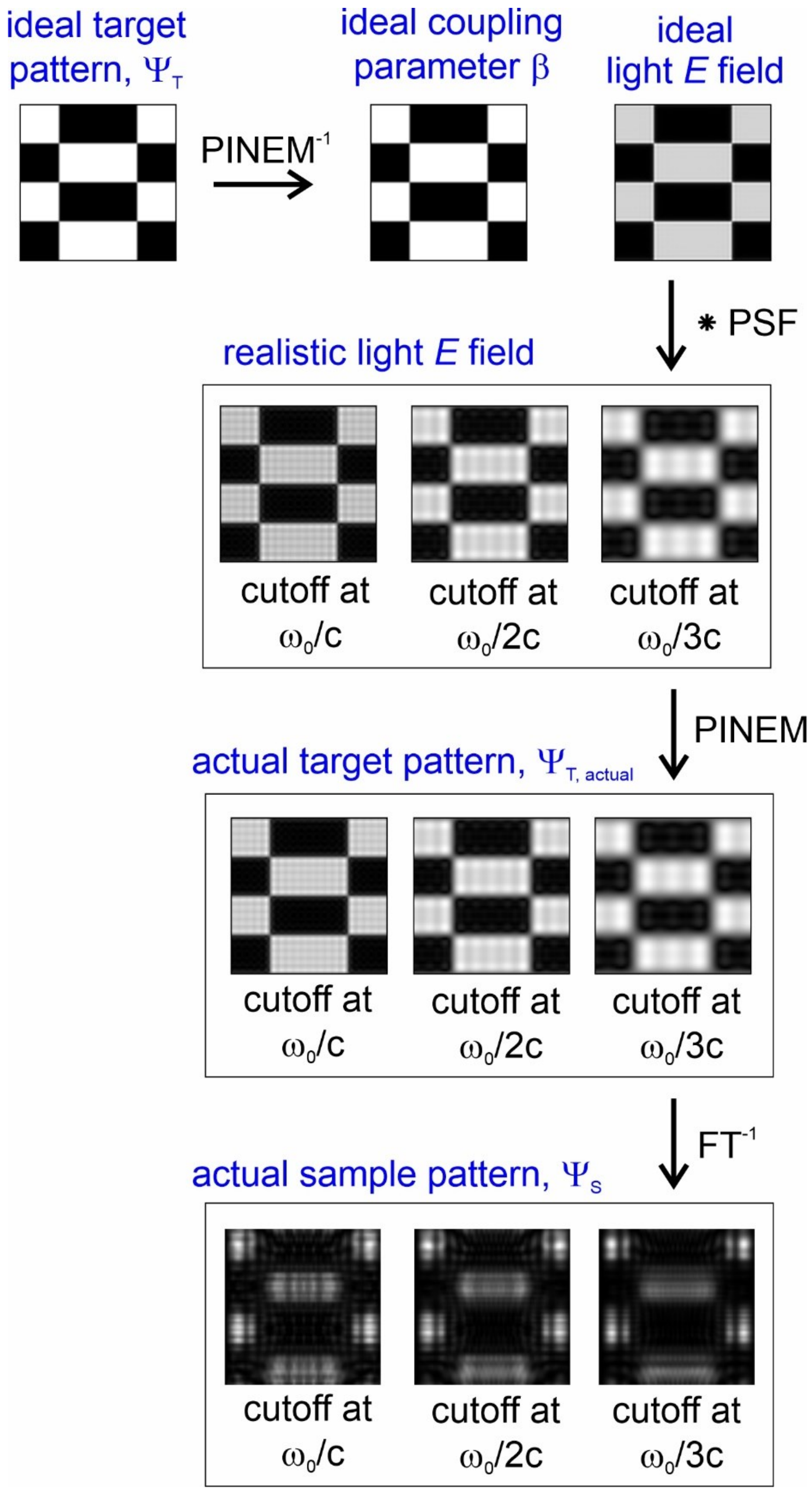

Figure S1. Sequence of operations used to calculate the transverse distribution of beam electrons arriving on the sample when starting from an ideal target pattern and considering realistic non-ideal conditions. Here, we plot results for a pattern taken from a Hadamard basis.

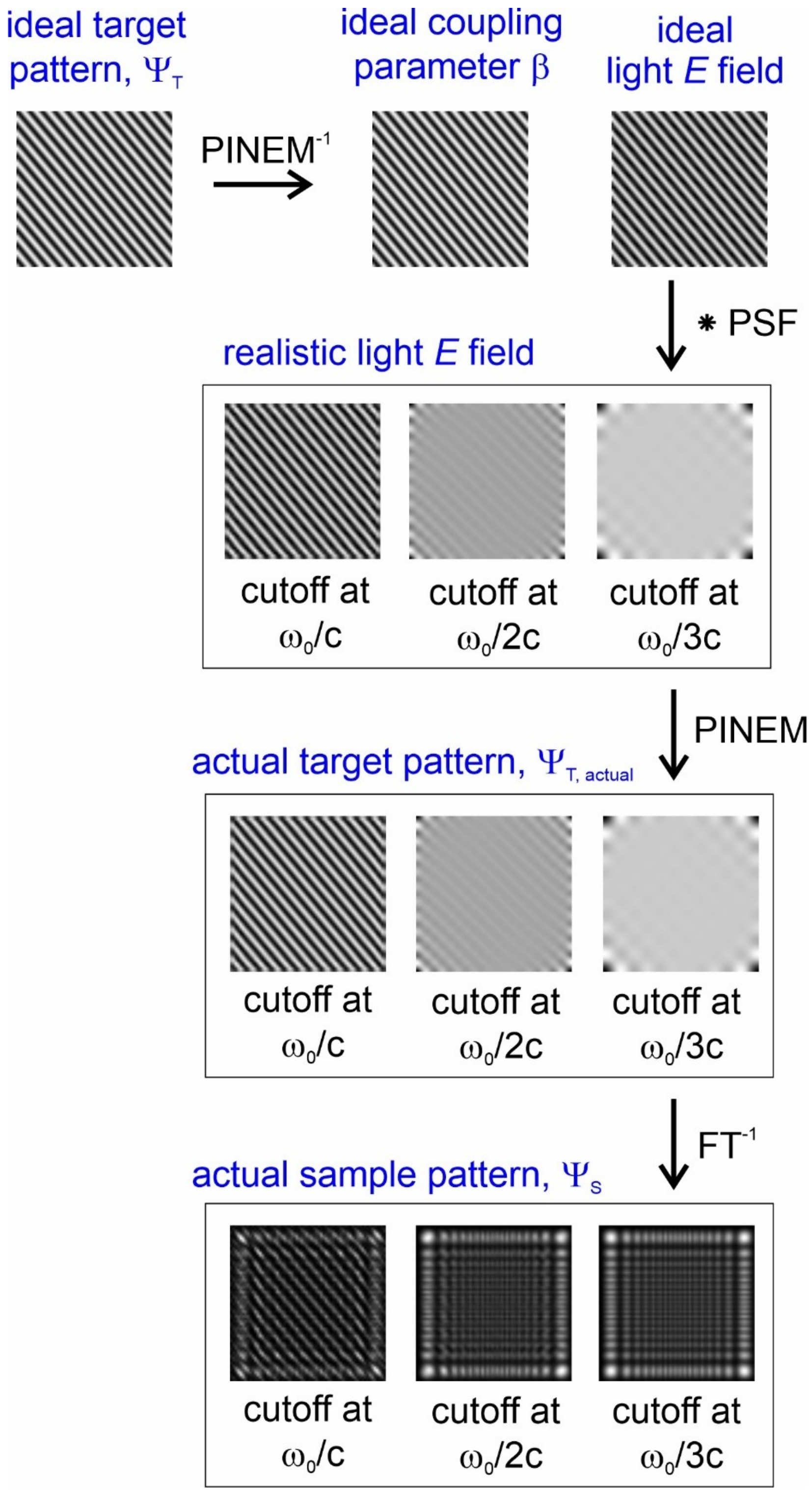

Figure S2. Sequence of operations used to calculate the transverse distribution of beam electrons arriving on the sample when starting from an ideal target pattern and considering realistic non-ideal conditions. Here, we plot results for a pattern taken from a Fourier basis.

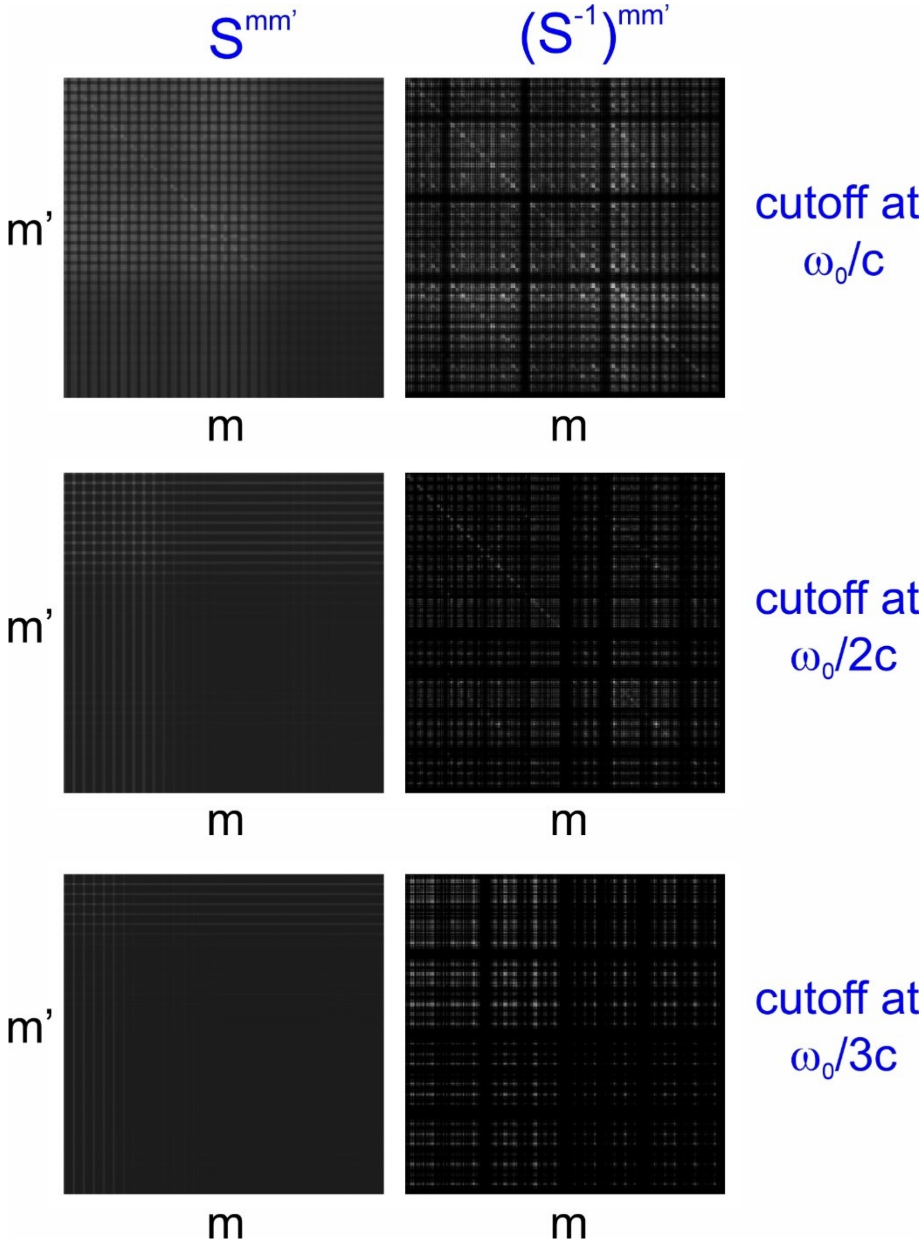

Figure S3. Overlap matrix calculated for the Hadamard basis when considering imperfect illumination of the sample plotted for the three different cutoffs considered in this work.
